# IUC26666-91 Prediction of immune-related adverse events (irAEs) with hematologic markers in patients receiving first-line immune checkpoint inhibitor (ICI)-based therapy in metastatic renal cell carcinoma (mRCC)

**DOI:** 10.1093/oncolo/oyag286.018

**Published:** 2026-08-21

**Authors:** R Winayak, X Li, N J Salgia, K Makins, V A De Goes, A Moradi, J Hsu, H Ebrahimi, A Chehrazi-Raffle, S K Pal

**Affiliations:** Department of Medical Oncology, City of Hope Comprehensive Cancer Center, Duarte, CA - United States; Department of Medical Oncology, City of Hope Comprehensive Cancer Center, Duarte, CA - United States; Department of Immunology, Roswell Park Comprehensive Cancer Center, Buffalo, NY - United States; Department of Medical Oncology, City of Hope Comprehensive Cancer Center, Duarte, CA - United States; Department of Medical Oncology, City of Hope Comprehensive Cancer Center, Duarte, CA - United States; Department of Medical Oncology, City of Hope Comprehensive Cancer Center, Duarte, CA - United States; Department of Medical Oncology, City of Hope Comprehensive Cancer Center, Duarte, CA - United States; Beth Israel Deaconess Medical Center, Boston, MA - United States; Department of Medical Oncology, City of Hope Comprehensive Cancer Center, Duarte, CA - United States; Department of Medical Oncology, City of Hope Comprehensive Cancer Center, Duarte, CA - United States

## Abstract

**Background:**

First-line therapies with combinations of ICI-ICI and ICI-tyrosine kinase inhibitor (TKI) are commonplace in mRCC. The frequency of irAEs in patients receiving first-line ICI-based therapies is high, with grade ≥ 2 (G ≥ 2) typically warranting active management. Although prognostication based on irAEs has been demonstrated, the prediction of irAE occurrence in mRCC remains a challenge. We investigated the role of baseline hematologic markers to predict G ≥ 2 irAE occurrence with first-line ICI-based therapies in mRCC.

**Methods:**

We used the City of Hope database (encompassing 5 centers across 4 states) to retrospectively identify mRCC patients treated with first-line ICI-based therapies from September 2015 to September 2025. Baseline patient demographics, including age, gender, Karnofsky Performance Status (KPS), pre-existing autoimmune condition, IMDC risk group and first-line ICI-based regimen, were collected. We stratified baseline hematologic markers, including neutrophil-to-lymphocyte ratio (NLR), neutrophil-to-eosinophil ratio (NER), platelet-to-lymphocyte ratio (PLR) and systemic inflammatory index (SII) into high and low levels using recursive partitioning, and evaluated their association with the occurrence of first-line ICI-related G ≥ 2 irAEs.

**Results:**

A total of 198 patients with mRCC were treated with first-line ICI-based therapy, of which 5 were excluded due to insufficient data. Of the 193 evaluable patients, the median age was 64.2, 131 patients (67.9%) were male, 100 patients (51.8%) received ICI-ICI, and 93 patients (48.2%) received ICI-TKI. G ≥ 2 irAEs were observed in 55 patients (28.5%), requiring systemic steroid use in 32 patients (16.6%) and treatment discontinuation in 30 patients (15.5%). The most common G ≥ 2 irAE was ALT/AST elevations, seen in 18 patients (9.3%). Of the baseline hematologic markers studied, increased PLR and SII were most strongly associated with the occurrence of G ≥ 2 irAEs (P < 0.005 for each).

**Conclusions:**

Our results point to a novel potential role of PLR and SII as predictive biomarkers of G ≥ 2 irAEs, with ICI-based therapies in mRCC, meriting further validation in prospective studies.

